# From famine to NAFLD: long-term association between fetal famine exposure and adult non-alcoholic fatty liver disease

**DOI:** 10.3389/fmed.2026.1947597

**Published:** 2026-09-10

**Authors:** Xiaoli Zhang, Peiwen Dong, Minfang Lian, Lin Li

**Affiliations:** The Third People's Hospital of Chengdu, Chengdu, Sichuan, China

**Keywords:** early life, health examination, malnutrition, NAFLD, the Great Chinese Famine

## Abstract

**Background:**

Malnutrition in early life may change the structure and function of the body, which lead to a number of diseases in adulthood. There are very few studies in health examination population about the prevalence of NAFLD (non-alcoholic fatty liver disease) in adulthood after early experiences of malnutrition.

**Methods:**

A total of 4,188 participants were selected as research objects from a large tertiary Class A general hospital in western China. We divided the subjects into four groups based on their ages at the time of exposure to the famine during the era of the Great Chinese Famine. Binary logistic regression models were used to examine the associations of famine exposure with prevalent mild and moderate to severe NAFLD. Model 1 was adjusted for gender and age, while Model 2 further controlled for overweight/obesity, elevated blood glucose, elevated blood pressure and dyslipidemia.

**Results:**

Fetal famine exposure was significantly associated with higher prevalence of both mild and moderate to severe NAFLD compared with the non famine-exposed group (*P* < 0.05). No statistically significant differences were observed in the risk of mild or moderate to severe NAFLD between the early-childhood exposure group, middle-childhood exposure group and the non famine-exposed group (*P* > 0.05). Sensitivity analyses restricted to the 1959-1961 birth cohort (*N* = 291) further confirmed the significant association between fetal famine exposure and NAFLD. We also conducted sensitivity analyses using adjacent birth cohorts. In adjacent cohort 1 (born 1955-1958, *N* = 487), Model 2-adjusted ORs were 1.33 (95%CI: 0.90-1.94) for mild NAFLD and 0.95 (95%CI: 0.63-1.44) for moderate to severe NAFLD versus the non famine-exposed group. For adjacent cohort 2 (born 1963-1966, *N* = 908), corresponding Model 2 ORs were 1.39 (95%CI: 0.97-1.99) and 1.38 (95%CI: 0.96-2.00) for mild and moderate to severe NAFLD, respectively. These adjacent- cohort sensitivity analyses showed no statistically significant associations between famine exposure and NAFLD (all *P* > 0.05), supporting the time-window-specific nature of exposure associations.

**Conclusion:**

Fetal famine exposure may be associated with the development of adult NAFLD.

## Introduction

In recent decades, epidemiological research has focused on how environmental factors affect health (1–3). The Great Chinese Famine (1959-1962) is a key historical event studied for its lasting health impacts (4, 5). This famine caused immediate deaths and significantly influenced the health of individuals exposed during crucial developmental stages, like *in utero* or childhood (6, 7). Understanding these long-term associations are vital, particularly concerning the rise of metabolic disorders such as NAFLD (non-alcoholic fatty liver disease), obesity and diabetes in these years. The prevalence of NAFLD is increasing world wide, with nearly 1 billion people globally affected which represents a heavy clinical and economic burden on society. Severe NAFLD can progress to cirrhosis and hepatocellular carcinomas, leading eventually to organ failure. Despite this worrisome development, no approved pharmaco- therapies are currently available to treat this disease (8, 9). Therefore, it is critical to understand the factors influencing the development of NAFLD.

Current literature highlights a concerning association between early-life nutritional deprivation and an increased risk of metabolic syndromes later in life (10, 11). Extensive epidemiological studies have demonstrated that individuals exposed to famine during fetal development exhibit a significantly elevated risk of developing metabolic disorders, including obesity, hypertension, and dyslipidemia (12–14). This phenomenon is believed to originate from the body's adaptive responses to scarcity, which may lead to metabolic programming that renders individuals more susceptible to various chronic diseases when they are subsequently exposed to an obesogenic environment (15, 16). Although there is growing evidence linking nutritional stress in early life to adverse health outcomes, there are very few relevant studies in specific populations undergoing active health screening. Health-check participants represent a large real-world subgroup who routinely receive abdominal ultrasonography, enabling early detection of hepatic steatosis at relatively mild stages before clinical complications emerge (17). Compared with hospital-admitted patients or community cohorts, this population largely consists of adults with subclinical metabolic abnormalities, providing a unique opportunity to explore modifiable risk factors for NAFLD.

The present study subject was derived from the health-examination center of a large tertiary hospital in a provincial capital of Western China. We aimed to explore the relationship between the prevalence of NAFLD and famine exposure across distinct groups stratified by their age at the time of exposure to the Great Chinese Famine. Clinically, moderate to severe NAFLD carries higher risks of progression to steatohepatitis, fibrosis and adverse liver-related outcomes compared with mild NAFLD. Therefore, the outcome observed variables in the present study were classified into two grades: mild fatty liver and moderate to severe fatty liver. Critical windows of early-life nutritional deprivation exert distinct long-term metabolic influences depending on the developmental stage when undernutrition occurs. Consistent with widely adopted grouping strategies from previous famine-related studies, we stratified participants into four groups: those exposed to famine *in utero*, early-childhood, middle-childhood, and non-exposed controls (18, 19). This approach is grounded in the theoretical framework that emphasizes the critical windows of development and their potential impact on long-term health outcomes.

In conclusion, the research aims to enhance our understanding of the long-term associations related to early-life nutritional stress, thus informing public health strategies designed to mitigate these risks in vulnerable populations.

## Materials and methods

### Study subjects

**Figure 1:**
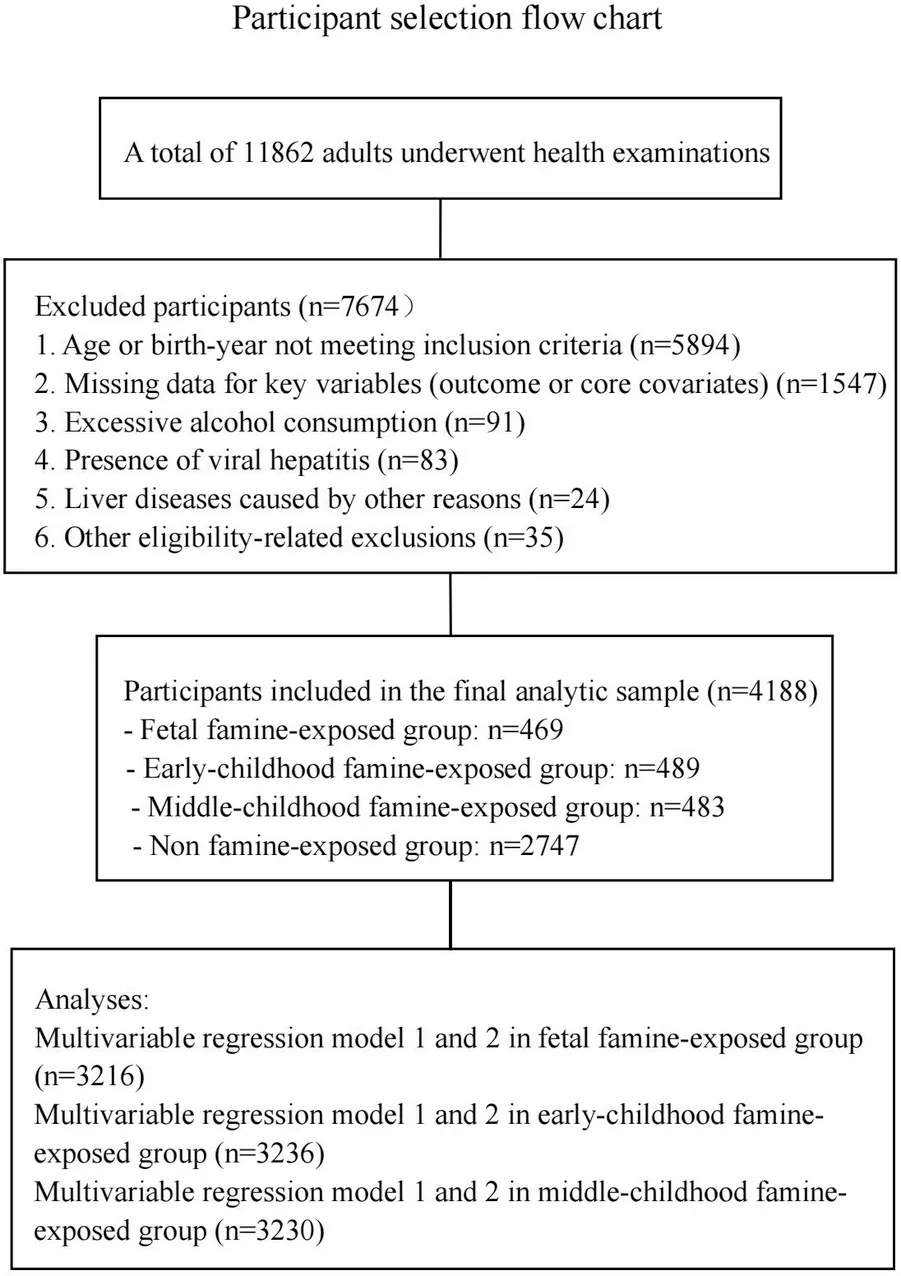
Adapted PRISMA flow diagram of participant selection for the retrospective cohort study. The figure summarizes the study-participant screening procedure according to pre-defined inclusion and exclusion criteria. Numbers of individuals excluded at each step and the final analytical sample are presented.

This study was conducted among individuals from the health examination center of a large tertiary Class A general hospital in a provincial capital city in western China. The study subjects were selected from 11,862 adults (aged >18 years) who underwent health check-ups at the center in 2024, including both males and females. These individuals exhibited no obvious symptoms or signs at that time and voluntarily participated in medical examinations to evaluate their health status. The study subjects included employees of state-owned enterprises, private enterprises, and self-employed individuals, covering various sectors such as government, healthcare, education, railways, and commerce. We have constructed a PRISMA-style flow chart (Figure 1) to clearly illustrate all exclusion steps, including exclusions due to age/birth year restriction, missing key variables and other eligibility criteria. Exclusion criteria for research subjects: history of excessive pure alcohol intake (≥40 g/day for men and≥20 g/day for women), viral liver diseases, and liver diseases caused by other reasons. Finally, A total of 4,188 subjects were included in this study. For missing data handling, complete-case analysis was applied. Participants with missing values for outcome, or core confounding covariates were excluded from the analysis. No multiple imputation was performed in the present study.

### Grouping of the research subjects

The Great Chinese Famine occurred from January 1959 to December 1962. In a cohort of 4,188 individuals undergoing physical examinations, exposure to famine was assessed based on their birth years. Following the research by Chen C (4), Meng R (5), et al., we categorized subjects into four groups according to their age during famine exposure:

- Fetal famine-exposed group (aged 62-65 years, born 1959–1962) (*n* = 469).
- Early-childhood famine-exposed group (aged 66–69 years, born 1955–1958) (*n* = 489)
- Middle-childhood famine-exposed group (aged 70–75 years, born 1949–1954) (*n* = 483).
- Non famine-exposed group (aged 50–61 years, born 1963–1974) (*n* = 2747).

### Variable definitions

Ultrasound is the primary imaging method for screening and diagnosing fatty liver. It is performed by certified B-ultrasound specialists. These specialists were unaware of the subjects' hepatic function status or the presence of fatty liver. They utilize Mindray M7 ultrasound equipment equipped with low-frequency probes for liver examinations. Normal: No hepatic steatosis. The liver parenchymal echoes have a similar intensity to renal echoes, and the hepatic vessels and diaphragm are clear; Mild fatty liver: Mild, diffuse increase in hepatic parenchymal echo points, with normal vascular and diaphragmatic clarity; Moderate to severe fatty liver: Ultrasound findings show diffuse and significantly enhanced liver echoes (markedly exceeding the levels of the renal and splenic echoes), accompanied by a pronounced contrast between hepatic and renal echoes and varying degrees of deep echo attenuation (moderate to severe attenuation or even disappearance of the posterior structure) (20, 21).

Weight measurement: Measured using a medical scale. Participants should remove outerwear and shoes. The measurement accuracy is up to 0.1 kg. Body Mass Index: BMI = Weight/Height (2). Here, Weight is measured in kilograms, and Height is measured in meters. A BMI between 24.0-27.9 indicates overweight, and a BMI ≥ 28 signifies obesity.

Elevated blood pressure: this encompasses two scenarios. First, systolic blood pressure≥140mmHg or diastolic blood pressure≥90mmHg without taking antihyper tensive medication. Second, individuals with a history of hypertension who are currently using antihypertensive drugs but have blood pressure levels below these thresholds are still classified as having elevated blood pressure.

Elevated blood glucose: Laboratory-defined elevated blood glucose refers to fasting blood glucose>6.1 mmol/L (fasting requires no caloric intake for at least 8 h), glycated hemoglobin ≥ 6.5%, or prior diabetes mellitus.

Abnormal lipid levels: Laboratory-defined lipid abnormalities include total cholesterol>5.72 mmol/L, triglycerides>1.70 mmol/L, LDL cholesterol > 3.10 mmol/L, or HDL cholesterol < 1.55 mmol/L.

### Statistical methods

We conducted statistical analysis using IBM SPSS 23.0 (IBM Corporation). Categorical variables in the study were presented as percentages. Logistic regression was used to examine the relationship between famine exposure and adult-onset fatty liver disease among different age groups. All statistical analyses used two-tailed tests, with *P* < 0.05 considered statistically significant.

## Results

### Basic characteristics of the study subjects

A total of 4,188 participants were included in the present study, including 469 individuals in the fetal famine exposure group, 489 in the early-childhood famine exposure group, 483 in the middle-childhood famine exposure group, and 2,747 in the non-exposed group. Baseline characteristics of all participants across the four groups are summarized in Table 1. There were differences in gender composition, age distribution, and metabolic indicators among the four groups. In the fetal famine exposure group, the prevalence of mild NAFLD was 32.4%, and the prevalence of moderate to severe NAFLD was 30.1%.

**Table 1:** Basic characteristics of research subjects in each group.

Characteristics	Fetal exposure group (*n* = 469)	Early-childhood exposure group (*n* = 489)	Middle-childhood exposure group (*n* = 483)	Non famine-exposed group (*n* = 2,747)	*P*-value
Age (years)	63.3 ± 1.2	67.6 ± 1.1	72.0 ± 1.6	55.2 ± 3.5	<0.001
Men, *n* (%)	293 (62.5)	285 (58.3)	271 (56.1)	1,359 (49.5)	<0.001
Women, *n* (%)	176 (37.5)	204 (41.7)	212 (43.9)	1,388 (50.5)	<0.001
Mild NAFLD, *n* (%)	152 (32.4)	141 (28.8)	147 (30.4)	364 (13.3)	<0.001
Moderate to severe NAFLD, *n* (%)	141 (30.1)	140 (28.6)	144 (29.8)	308 (11.2)	<0.001
Overweight/ Obesity, *n* (%)	214 (45.6)	194 (39.7)	227 (47.0)	814 (29.6)	<0.001
Elevated blood glucose, *n* (%)	106 (22.6)	118 (24.1)	124 (25.7)	228 (8.3)	<0.001
Elevated blood pressure, *n* (%)	198 (42.2)	219 (44.8)	269 (55.7)	544 (19.8)	<0.001
Dyslipidemia, *n* (%)	346 (73.8)	343 (70.1)	308(63.8)	1,418(51.6)	<0.001

Continuous variables are expressed as mean ± SD, and categorical variables as *n* (%). *P*-values were calculated using ANOVA (for age) or the chi-square test (for categorical variables).

### Association between famine exposure and NAFLD

Table 2 presents the logistic regression results for the associations of fetal-phase, early-childhood, and middle-childhood famine exposure with the prevalence of mild and moderate to severe NAFLD. In Model 1 (adjusted for gender and age), fetal famine exposure was significantly associated with elevated odds of both mild NAFLD (OR =2.20, 95%CI: 1.59-3.04, *P* < 0.001) and moderate to severe NAFLD (OR=2.11, 95%CI: 1.50-2.97, *P* < 0.001) relative to the non famine-exposed group. In Model 2 (adjusted for gender, age, overweight/obesity, elevated blood glucose, elevated blood pressure and dyslipidemia), fetal famine exposure remained independently associated with a higher risk of mild NAFLD (OR=1.90, 95%CI: 1.36-2.65, *P* < 0.001) and moderate to severe NAFLD (OR = 1.65, 95%CI: 1.14-2.38, *P* = 0.007). For participants with early-childhood famine exposure, a significant crude association was observed for mild NAFLD in Model 1 (OR = 1.63, 95%CI: 1.06-2.51, *P* = 0.026), while the association became non-significant after further adjustment in Model 2 (OR = 1.49, 95%CI: 0.96-2.31, *P* = 0.076). The association for moderate to severe NAFLD was marginally significant in Model 1 (*P* = 0.051) and turned non-significant in Model 2 (*P* = 0.228). No statistically significant associations were detected for middle- childhood famine exposure across both regression models (all *P* > 0.05).

**Table 2:** Binary logistic regression analysis of the prevalence of NAFLD.

Subgroups	Model 1		Model 2	
	OR(95%CI)	*P*-value	OR(95%CI)	*P*-value
Fetal exposure group (*N* = 469)				
Mild NAFLD	2.20 (1.59–3.04)	<0.001	1.90 (1.36–2.65)	<0.001
Moderate to severe NAFLD	2.11 (1.50–2.97)	<0.001	1.65 (1.14–2.38)	0.007
Early-childhood exposure group (*N* = 489)				
Mild NAFLD	1.63 (1.06–2.51)	0.026	1.49 (0.96–2.31)	0.076
Moderate to severe NAFLD	1.57 (1.00–2.47)	0.051	1.35 (0.83–2.19)	0.228
Middle-childhood exposure group (*N* = 483)				
Mild NAFLD	1.60 (0.92–2.75)	0.094	1.44 (0.82–2.53)	0.202
Moderate to severe NAFLD	1.35 (0.76–2.40)	0.309	1.11 (0.60–2.07)	0.739
Non famine- exposed group (*N* = 2,747)				
Mild NAFLD	1.00(Ref)	-	1.00(Ref)	-
Moderate to severe NAFLD	1.00(Ref)	-	1.00(Ref)	-

Model 1: adjusting for gender and age.

Model 2: adjusting for gender, age, overweight/obesity, elevated blood glucose, elevated blood pressure and dyslipidemia.

### Sensitivity analysis: 1959–1961 birth cohort

To verify the time-window specificity of fetal famine exposure, we restricted the fetal-exposed subgroup to the 1959-1961 birth cohort (*N* = 291) for comparison with the non-exposed group. In this subgroup, the OR for mild NAFLD was 2.54 (95%CI: 1.70-3.79, *P* < 0.001), and the OR for moderate to severe NAFLD was 1.54 (95%CI: 1.01-2.35, *P* = 0.044) in Model 2. These findings further support the association between fetal famine exposure and NAFLD. These results are presented in Table 3.

**Table 3:** Sensitivity analysis of the association between the 1959-1961 birth cohort and NAFLD.

Subgroups	Model 1		Model 2	
	OR(95%CI)	*P*-value	OR(95%CI)	*P*-value
Birth: 1959–1961 (*N* = 291)				
Mild NAFLD	2.93 (1.98–4.34)	<0.001	2.54 (1.70–3.79)	<0.001
Moderate to severe NAFLD	2.05 (1.39–3.01)	<0.001	1.54 (1.01–2.35)	0.044
Non famine- exposed group (*N* = 2,674)				
Mild NAFLD	1.00(Ref)	-	1.00(Ref)	-
Moderate to severe NAFLD	1.00(Ref)	-	1.00(Ref)	-

Model 1: adjusting for gender and age.

Model 2: adjusting for gender, age, overweight/obesity, elevated blood glucose, elevated blood pressure and dyslipidemia.

### Adjacent cohort sensitivity analysis

To rule out confounding by birth-cohort heterogeneity, we performed sensitivity analyses using adjacent birth cohorts as pseudo-exposed groups. For adjacent cohort 1 (born 1955-1958, *N* = 487), compared with the non famine-exposed group, Model 1 yielded an OR of 1.40 (95%CI: 0.96-2.03, *P* = 0.080) for mild NAFLD and 1.09 (95%CI: 0.74-1.60, *P* = 0.672) for moderate to severe NAFLD. After further adjustment in Model 2, the corresponding ORs were 1.33 (95%CI: 0.90-1.94, *P* = 0.148) for mild NAFLD and 0.95 (95%CI: 0.63-1.44, *P* = 0.804) for moderate to severe NAFLD. For adjacent cohort 2 (born 1963-1966, *N* = 908), in Model 1, the OR was 1.55 (95%CI: 1.09-2.21, *P* = 0.014) for mild NAFLD and 1.70 (95%CI: 1.21-2.39, *P* = 0.002) for moderate to severe NAFLD. Following additional adjustment in Model 2, the ORs were 1.39 (95%CI: 0.97-1.99, *P* = 0.073) for mild NAFLD and 1.38 (95%CI: 0.96-2.00, *P* = 0.081) for moderate to severe NAFLD. Neither of the two adjacent cohorts exhibited statistically significant associations with NAFLD, supporting the time-window specificity of fetal famine exposure associations. These results are presented in Table 4.

**Table 4:** Sensitivity analysis of the association between adjacent cohorts and NAFLD.

Subgroups	Model 1		Model 2	
	OR(95%CI)	*P*-value	OR(95%CI)	*P*-value
Adjacent cohort 1 (1955–1958)				
Mild NAFLD	1.40 (0.96–2.03)	0.080	1.33 (0.90–1.94)	0.148
Moderate to severe NAFLD	1.09 (0.74–1.60)	0.672	0.95 (0.63–1.44)	0.804
Adjacent cohort 2 (1963–1966)				
Mild NAFLD	1.55 (1.09–2.21)	0.014	1.39 (0.97–1.99)	0.073
Moderate to severe NAFLD	1.70 (1.21–2.39)	0.002	1.38 (0.96–2.00)	0.081
Non famine- exposed group				
Mild NAFLD	1.00(Ref)	-	1.00(Ref)	-
Moderate to severe NAFLD	1.00(Ref)	-	1.00(Ref)	-

Model 1: adjusting for gender and age.

Model 2: adjusting for gender, age, overweight/obesity, elevated blood glucose, elevated blood pressure and dyslipidemia.

## Discussion

Human life can be divided into the early, middle and late stages (22). Currently, it remains unclear whether adverse events in early life are associated with the onset and progression of non-communicable diseases in later life. There was a DOHaD theory suggesting that in addition to lifestyle and genetic inheritance in adulthood, the nutritional status in the early stage of life can also affect the risk of certain adult non-communicable diseases, especially metabolic-related diseases (23, 24). However, the nationwide Great Chinese Famine from 1959 to 1962 may represent one of the critical influencing factors. This study researched the association between exposure to the great famine era in China in early life and the prevalence of NAFLD in adulthood.

NAFLD is a clinico-pathological syndrome primarily characterized by hepatic steatosis in the absence of alcohol consumption or other well-defined causes of liver injury. NAFLD can progress to liver cirrhosis and even liver cancer in severe cases. NAFLD is often associated with metabolic syndrome, obesity, insulin resistance, and dyslipidemia (25–27). In western countries and many asian nations, changes in diet and lifestyle have led to a significant increase in the incidence of obesity and metabolic syndrome, as well as a marked rise in the incidence of NAFLD. NAFLD has now become the most common liver disease in both China and Western countries, and is one of the important public health issues worldwide in the 21st century (28, 29). The growing burden of NAFLD highlights the urgent need for effective screening and management strategies to mitigate its impact on liver-related morbidity and mortality (30), particularly in populations exposed to historical nutritional adversities, such as the Great Chinese Famine, which may have long-lasting associations on liver health and metabolic conditions in subsequent generations.

In the present study, we investigated the association between famine exposure during critical developmental periods and the prevalence of NAFLD. By categorizing subjects based on their exposure to famine during fetal, early-child hood and middle-childhood stages, we assessed the prevalence and severity of hepatic steatosis using ultrasound, alongside evaluating other metabolic parameters such as overweight/obesity, hypertension, and dyslipidemia among others. Binary logistic regression showed that fetal famine exposure was independently associated with mild and moderate to severe NAFLD after further adjustment for confounders in Model 2. The crude association for early-childhood famine exposure vanished after metabolic adjustment, while middle-childhood exposure showed no significant links. Adjacent-birth-cohort sensitivity analyses produced heterogeneous findings: no associations were found using the 1955-1958 reference cohort, and crude significance in the 1963-1966 group disappeared after covariate adjustment. These results partly support the time-window-specific nature of exposure-related associations. First, Baseline imbalance and birth-cohort heterogeneity may inflate crude association estimates. The 1955-1958 cohort experienced early-life food shortage, sharing similar metabolic profiles with the exposed group and weakening the association; the post-famine 1963-1966 cohort had better childhood nutrition, yielding detectable crude risk before metabolic adjustment (31, 32). Second, selective survival bias is a key limitation of famine epidemiological research. Early-life malnutrition may reduce survival among frail individuals, generating a healthier surviving population with lower metabolic disease risk in adulthood (33). This survival selection attenuates detectable long-term famine associations and partly accounts for our null findings or attenuated findings in fully adjusted models, consistent with systematic methodological reviews (34). Third, heterogeneity across famine literature largely originates from variations in exposure definition, regional famine severity assessment, and control group selection (35). In contrast, the present study applied strict cohort criteria and adjacent birth year sensitivity analyses to obtain conservative association estimates. It should be noted that gender and age were pre-exposure confounders and adjusted uniformly in Model 1 for this study. overweight/obesity, elevated blood glucose, elevated blood pressure and dyslipidemia may be downstream consequences of early-life famine exposure which represent post-exposure mediators. Adjustment for these mediators in Model 2 carries a risk of over-adjustment, which may attenuate effect estimates. Nevertheless, the consistently positive association for fetal famine exposure even after metabolic adjustment indicates the robustness of this finding. Overall, only fetal famine exposure retained an independent association with NAFLD after full adjustment, suggesting the intrauterine period as a critical window for long-term hepatic metabolic programming.

The present study provides valuable insights into the long-term associations of famine exposure during critical developmental periods, particularly in relation to the prevalence of NAFLD among adults in a specific population in western China. Evidence from multiple historical famine cohorts provides important insights into the long-term health consequences of prenatal nutritional adversity. The Dutch Potato Famine of 1846-1847 demonstrated that early-life nutritional shock was associated with reduced longevity in later life, though metabolic liver outcomes were not assessed in that work (36). Research on the Dutch Hunger Winter further revealed persistent metabolic programming following intrauterine undernutrition (37). Stein et al. observed long-term alterations in anthropometric parameters among individuals exposed to famine during gestation (38). A recent large-scale study by Lumey et al., based on the Ukrainian famine, reported a dose-response association between prenatal nutritional deprivation and adult-onset type 2 diabetes mellitus (39). Nevertheless, none of the above-mentioned studies evaluated non-alcoholic fatty liver disease. Differences in famine duration, dietary composition, post-famine socioeconomic environment, genetic background and adult lifestyle limit the direct generalisation of these European-based findings to Chinese populations. Accordingly, our retrospective analysis of health-check-up participants adds new population-level evidence regarding the association between fetal-life famine exposure and adult NAFLD within an East-Asian setting. Collectively, our study extends these implications to a large sample of employees from different fields undergoing active health screening, thereby adding valuable real-world evidence to existing literature concerning the long-term health impacts of historical famine exposure within this specific subgroup.

The clinical implications of our findings are important, as they underscore the need for targeted health interventions for individuals who were exposed to famine during critical growth periods. These individuals may benefit from early screening and preventive strategies for NAFLD and related metabolic disorders, thereby informing public health policies aimed at mitigating the long-term associations of nutritional deprivation. Furthermore, the observed increase in metabolic syndrome components among the fetal famine exposure group suggests a potential for increased healthcare costs and resource allocation in managing chronic diseases stemming from early-life nutritional stressors. As such, our findings advocate for a re-evaluation of health policies to incorporate a life-course perspective on health, emphasizing the importance of maternal and early childhood nutrition.

## Limitations

Although our study has several strengths, it has several main limitations as outlined below. Firstly, this is a single-center retrospective observational study. Therefore, causal relationships cannot be established merely based on the observed associations. Multi-center prospective cohorts are required to further validate our conclusions in the future. Secondly, all participants were derived from health check-up attendees at a tertiary hospital. The enrolled subjects may not be representative of the general population, creating potential selection bias. Access to routine hospital physical examinations is uneven across the population, which may distort the distribution of metabolic risk factors. Thirdly, socioeconomic covariates such as education level, occupation, household registration and marital status were not available. Without these data, residual confounding related to socioeconomic status cannot be completely excluded. Fourthly, regional differences in famine severity were not considered. Since participants' birthplace and residential location during the famine era were not verified, population migration may cause exposure misclassification and limit the accuracy of exposure assessment.

## Conclusion

In summary, this study suggests early-life famine exposure may be associated with elevated risk of adult NAFLD. The findings extend evidence for the DOHaD hypothesis in metabolic liver diseases. The above limitations should be considered when interpreting results, and further multicenter prospective studies are required for validation.

## Data Availability

The original contributions presented in the study are included in the article/Supplementary Material, further inquiries can be directed to the corresponding author.
